# The EIMS fragmentation mechanisms of the sesquiterpenes corvol ethers A and B, *epi*-cubebol and isodauc-8-en-11-ol

**DOI:** 10.3762/bjoc.12.132

**Published:** 2016-07-05

**Authors:** Patrick Rabe, Jeroen S Dickschat

**Affiliations:** 1 Kekulé-Institute of Organic Chemistry and Biochemistry, University of Bonn, Gerhard-Domagk-Straße 1, 53121 Bonn, Germany

**Keywords:** bacteria, isotopic labelling, mass spectrometry, reaction mechanisms, terpenes

## Abstract

Farnesyl diphosphate (FPP) and all fifteen positional isomers of (^13^C_1_)FPP were enzymatically converted by the bacterial terpene cyclases corvol ether synthase from *Kitasatospora setae*, the *epi*-cubebol synthase from *Streptosporangium roseum*, and the isodauc-8-en-11-ol synthase from *Streptomyces venezuelae*. The enzyme products were analysed by GC–MS and GC–QTOF MS^2^ and the obtained data were used to delineate the EIMS fragmentation mechanisms of the two sesquiterpene ethers corvol ethers A and B, and the sesquiterpene alcohols *epi*-cubebol and isodauc-8-en-11-ol.

## Introduction

Gas chromatography coupled to electron impact mass spectrometry (GC–EIMS) is a powerful and broadly applied method to investigate volatile natural products in complex mixtures [1]. Positive compound identification requires a good match of both the measured mass spectrum and retention time to the corresponding data obtained from an authentic standard. Furthermore, various high quality databases containing the EI mass spectra and retention indices of thousands of compounds are available that assist in automated compound identification [2–3]. If unknown compounds are detected in natural extracts, their structure elucidation by GC–MS is more difficult. The profound knowledge about EIMS fragmentation reactions can be used to identify certain structural motifs, e.g., the mass spectra of acyclic carbonyl compounds are often dominated by fragment ions formed via McLafferty rearrangement [4], while cyclohexene derivatives often show major fragment ions produced via retro-Diels–Alder reaction [5–6]. Such diagnostic fragment ions are of high value to delineate structural proposals for unknown analytes from their mass spectra, but for unambiguous proof of the suggested structures a synthesis of reference material is essential. Back in the 1960s, Ryhage and Stenhagen presented detailed studies on the EI mass spectra of deuterated and methyl-branched fatty acid methyl esters that revealed their fragmentation mechanisms [7–8]. Based on this work, we have recently identified various volatile fatty acid methyl esters (FAMEs) in headspace extracts of the actinobacterium *Micromonospora aurantiaca* [9] and more than 30 blastmycinones, a class of γ-lactones that depend on the antimycin biosynthetic gene cluster in several streptomycetes [10].

We have also recently developed structural proposals for a series of methylated monoterpenes from the 2-methylisoborneol biosynthetic pathway by comparing the mass spectra of the methylated compounds to their non-methylated analogs [11]. Higher terpenes such as sesqui- and diterpenes, as being produced by terpene cyclases from oligoprenyl diphosphates, are usually (poly)cyclic compounds with different ring sizes, contain several methyl groups, and possibly one or more olefinic double bonds, an alcohol or ether function. Their much higher structural complexity compared to, e.g., FAMEs and monoterpenes renders a prediction of the fragmentation behaviour of unknown compounds in mass spectrometry and consequently the development of structural proposals from their mass spectra impossible. Only very few studies have addressed the fragmentation mechanisms of terpenes using isotopically labelled compounds [12–15], likely because the synthesis of the required labelled material is laborious and expensive. Furthermore, introduction of labelling into the various positions of the compound of interest may require a different synthetic strategy for each individual target isotopomer. Most of these studies made use of deuterium labellings that can frequently be introduced into reactive positions of a (functionalised) terpene isolated from the producing organism. Deuterium labellings also allow to follow hydrogen rearrangements, but non-specific hydrogen migrations during the fragmentation process and kinetic isotope effects can make data interpretation difficult. In contrast, the introduction of ^13^C-labelling into a terpene requires a de novo synthesis for each isotopomer, or at least a partial degradation of a terpene and reconstruction with a ^13^C-labelled building block. Alternatively, a ^13^C-labelled terpene may be obtained by feeding of labelled precursors to the producing organism, but this strategy will require high incorporation rates and usually delivers a mixture of various isotopomers. We have recently synthesised all fifteen isotopomers of (^13^C_1_)farnesyl diphosphate (FPP) that can be enzymatically converted with a sesquiterpene cyclase into the corresponding labelled sesquiterpene products [16]. These enzyme products carry a labelling (>99% ^13^C) in specific positions that can easily be located, if the cyclisation mechanism of the terpene cyclase is known. Furthermore, ^13^C NMR spectroscopy can be used to experimentally locate the labelling if the cyclisation mechanism is unidentified. As we have shown in two previous studies with (1(10)*E*,4*E*,6*S*,7*R*)-germacradien-6-ol synthase from *Streptomyces pratensis* and *epi*-isozizaene synthase from *Streptomyces albus*, that the enzymatically obtained products from the (^13^C_1_)FPP isotopomers are useful for detailed investigations on the EIMS fragmentation mechanisms of sesquiterpenes [16–17]. In the present study we have used the same approach to investigate the fragmentation mechanisms for corvol ethers A and B, two sesquiterpene ethers with unique carbon skeletons that are made by a terpene cyclase from *Kitasatospora setae* [18], and the sesquiterpene alcohols *epi*-cubebol and isodauc-8-en-11-ol made by terpene cyclases from *Streptosporangium roseum* [19–20] and from *Streptomyces venezuelae* [21].

## Results and Discussion

**Scheme 1:**
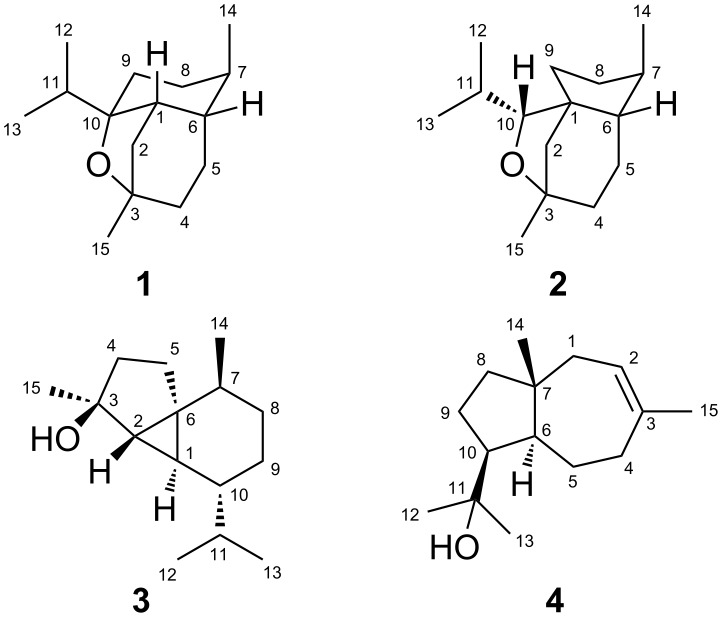
Structures of corvol ethers A (**1**) and B (**2**), *epi*-cubebol (**3**), and isodauc-8-en-11-ol (**4**). Carbon numbering is not systematic, but is the same as for FPP, indicating the biosynthetic origin of each carbon by identical numbering.

To investigate the EIMS fragmentation mechanisms for the two sesquiterpene ethers corvol ether A (**1**) and corvol ether B (**2**), and for the sesquiterpene alcohols *epi*-cubebol (**3**) and isodauc-8-en-11-ol (**4**) (Scheme 1), all fifteen positional isomers of (^13^C_1_)FPP [16] were enzymatically converted by the corvol ether synthase from *K. setae* KM-6054 [18], the *epi*-cubebol synthase from *S. roseum* DSM 43021 [19–20], and the isodauc-8-en-11-ol synthase from *S. venezuelae* ATCC 10712 [21], respectively. In previous work all used enzymes were mechanistically thoroughly studied, and therefore the locations of labellings in the obtained products are known [18–22]. The enzyme products were analysed via GC–MS, and for each of the ^13^C-labelled isotopomers of the investigated sesquiterpenes some of the fragment ions in their mass spectra clearly increased by +1 amu, indicating that the corresponding carbon atom contributes to the fragment ion, while for other fragment ions no such increase was observed, showing that the respective labelled carbon is cleaved off during the formation. Furthermore, if multiple mechanisms lead to distinct fragment ions with the same nominal mass, representing different parts of the analyte, labelling of a carbon that does not belong to all these fragment ions will only lead to a partially increased *m*/*z* peak by +1 amu in the mass spectrum. This is particularly true for the fragment ions in the low mass range that can be formed via multiple mechanisms, and thus these ions are not discussed in detail here. For the full analysis of a certain fragment ion *m*/*z* by comparison of all fifteen (^13^C_1_)-isotopomers of a sesquiterpene to the non-labelled counterpart we have recently introduced the term ‘position-specific mass shift analysis’ for *m*/*z* (PMA*_m_*_/_*_z_*) [17].

### EIMS fragmentation of corvol ether A

**Figure 1:**
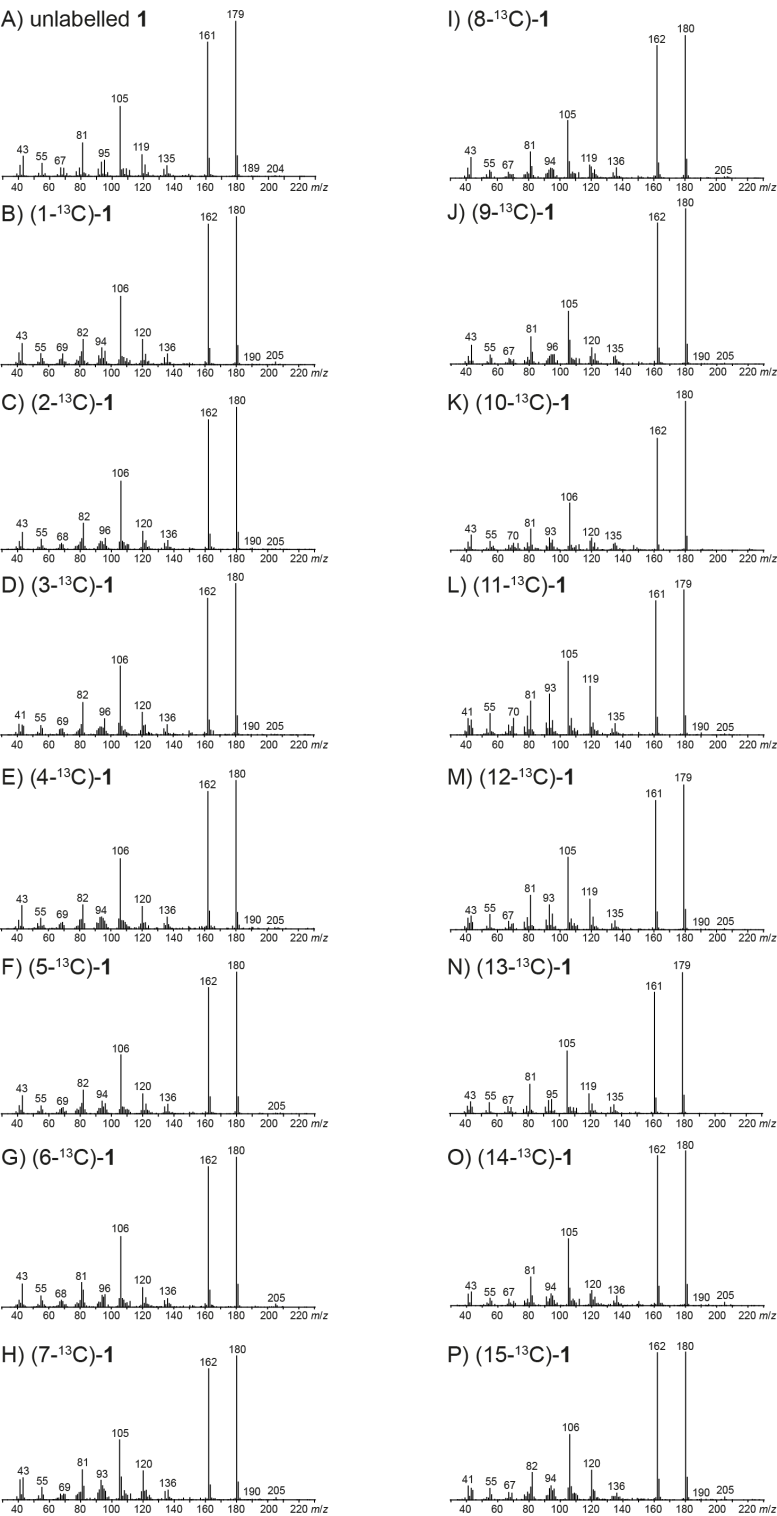
Mass spectra of unlabelled **1** and all fifteen positional isomers of (^13^C_1_)-**1**.

**Scheme 2:**
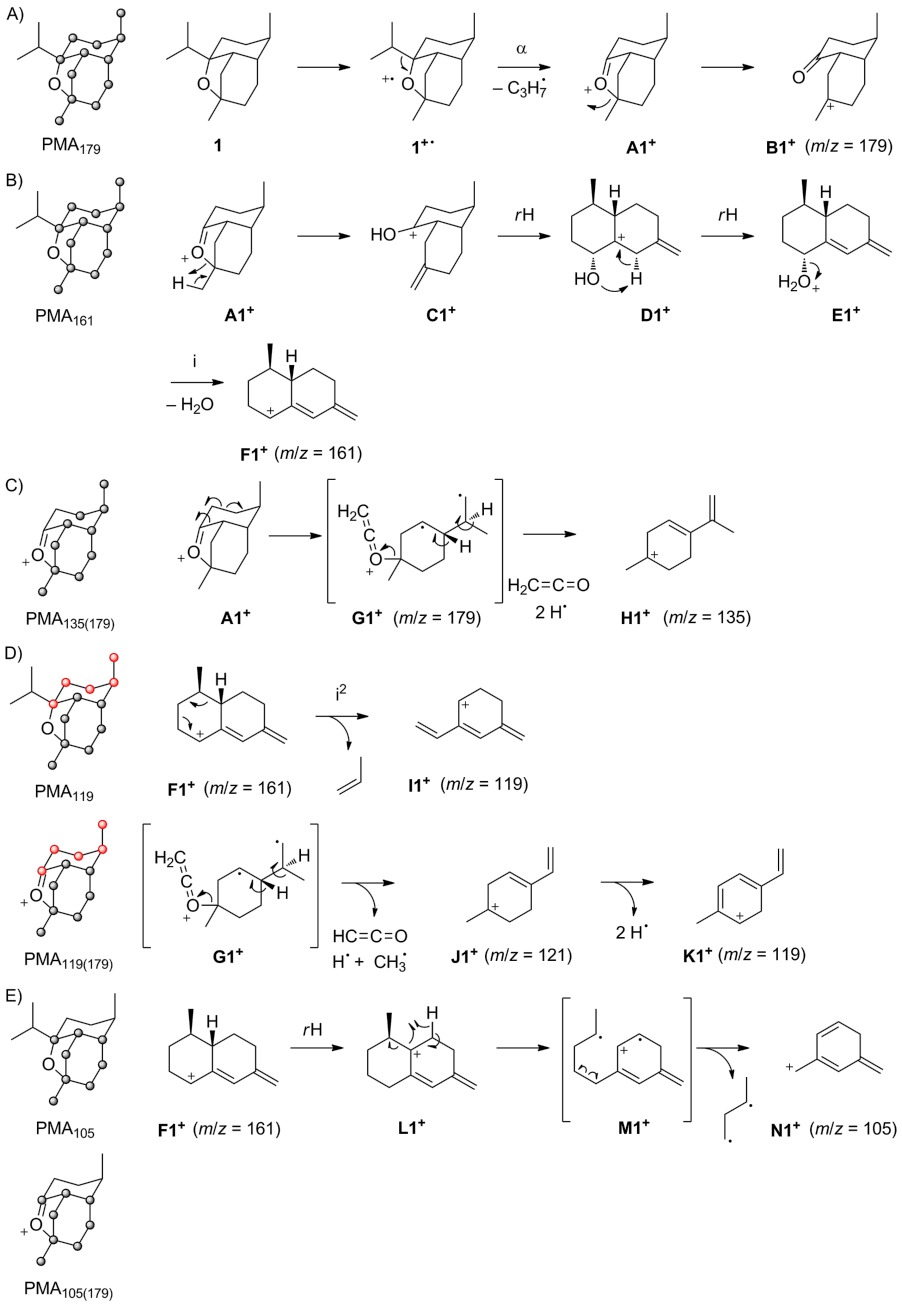
PMAs and EIMS fragmentation mechanisms for the fragment ions A) *m*/*z* = 179, B) *m*/*z* = 161, C) *m*/*z* = 135, D) *m*/*z* = 119 and E) *m*/*z* = 105 of **1**. Black carbons contribute fully and red carbons contribute partially to the formation of a fragment ion. α: α-cleavage, *r*H: hydrogen rearrangement, i: inductive cleavage.

The EI mass spectra of unlabelled **1** and all fifteen positional isomers of (^13^C_1_)-**1** are shown in Figure 1. The molecular ion [M]^+^, which is expected at *m*/*z* = 222 for unlabelled **1**, is not visible, suggesting a strong fragmentation of **1** upon electron impact ionisation. Besides the base peak at *m*/*z* = 179, major fragment ions are detected at *m*/*z* = 161 and *m*/*z* = 105. The position-specific mass shift analysis for *m*/*z* = 179 (PMA_179_) that summarises the extracted information from all the mass spectra in Figure 1 reveals a specific mechanism for the formation of the base beak ion with loss of the C11–C12–C13 portion of the molecule, while all other carbons contribute to this ion (marked in black, Scheme 2A). This is explainable by electron impact ionisation of **1** with loss of one electron from the oxygen lone pairs to **1^+·^**, followed by α-cleavage with loss of the isopropyl group to **A1^+^**. This cation may ring-open to the cation **B1^+^**. The PMA_161_ shows that the fragment ion *m*/*z* = 161 is made up from the same part of the carbon backbone of **1** (Scheme 2B). Furthermore, the high resolution GC–QTOF MS^2^ analysis of *m*/*z* = 179 reveals that the base peak ion is a direct precursor of *m*/*z* = 161 by the loss of water (Figure S1, Supporting Information File 1). Starting from cation **A1^+^**, a ring opening reaction with a proton transfer to the oxygen may yield **C1^+^** that upon hydrogen rearrangements via **D1^+^** to **E1^+^** and inductive cleavage of water results in the conjugated cation **F1^+^**. Since the ^13^C-labelling experiments presented in this study cannot distinguish which of the hydrogens of **1** are rearranged or lost, alternative mechanisms may contribute to the formation of *m*/*z* = 161, but such alternatives must end up in a fragment ion composed of the same carbon framework as for **F1^+^**.

Due to the low abundance of the fragment ion at *m*/*z* = 135 the PMA_135_ from the mass spectra in Figure 1 is not very conclusive, but seems to indicate a cleavage of the C9–C10 portion, besides the loss of C11–C12–C13 (Scheme 2C). MS^2^ analysis shows that this fragment ion arises from the base peak ion, but not from *m*/*z* = 161 (Figures S1 and S2, Supporting Information File 1). The fragmentation of C9–C10 was confirmed by MS^2^ analysis of *m*/*z* = 180 for all cases in which a ^13^C-labelling was present in **A1^+^** (Figure S3, Supporting Information File 1). Only for those isotopomers of **1** in which the ^13^C-labelling was located at C9 or C10 a fragment ion at *m*/*z* = 135, but no significant ion at *m*/*z* = 136 was detected, while for all other isotopomers a peak at *m*/*z* = 136 was observed (this method is abbreviated by PMA_135(179)_). The fragmentation of **A1^+^** may proceed via cleavage of two bonds to **G1^+^**, followed by loss of ketene via inductive cleavage and of two hydrogens by α-cleavage to yield **H1^+^**. The formation of diradicals that may be highly transient species and are shown in brackets in Scheme 2, can be avoided by the assumption of a concerted process from **A1^+^** to **H1^+^**, but it will be very difficult, if not impossible, to distinguish experimentally between a stepwise and the alternative concerted mechanism.

Both PMA_119_ and PMA_119(179)_ point to a formation of the ion *m*/*z* = 119 from the C1–C6 + C15 portion of **1** (Scheme 2D). Furthermore, two of the carbons C7–C10 + C14 participate in the formation of this fragment ion (carbons that only partially contribute to a fragment ion are marked in red in the PMAs), indicating that more than one fragmentation mechanism leading to fragment ions that represent different parts of **1** is active. Two inductive cleavages of **F1^+^** with neutral loss of propene result in **I1^+^**. Starting from **G1^+^**, a similar reaction as described above for **H1^+^** (*m*/*z* = 135) involves the neutral loss of ketene by inductive cleavage, and loss of one hydrogen and one methyl group by α-cleavages to produce **J1^+^** (*m*/*z* = 121). Finally, the loss of two more hydrogen atoms yields **K1^+^**.

PMA_105_ suggests a generation of the fragment ion *m*/*z* = 105 from C1–C6, C10 and C15 of **1** (Scheme 2E), while MS^2^ analysis confirms a strong formation of this fragment ion from *m*/*z* = 179 and 161 (Figures S1 and S2, Supporting Information File 1). Accordingly, all MS^2^ analyses for *m*/*z* = 180 of labelled **1** (Figure S3, Supporting Information File 1) indicate the origin of *m*/*z* = 105 from the same part of the carbon skeleton, as summarised by PMA_105(179)_. These findings are mechanistically explainable by a hydrogen rearrangement from **F1^+^** to **L1^+^**, followed by a ring opening with concomitant hydrogen rearrangement to **M1^+^** and α-cleavage to **N1^+^**.

### EIMS fragmentation of corvol ether B

**Figure 2:**
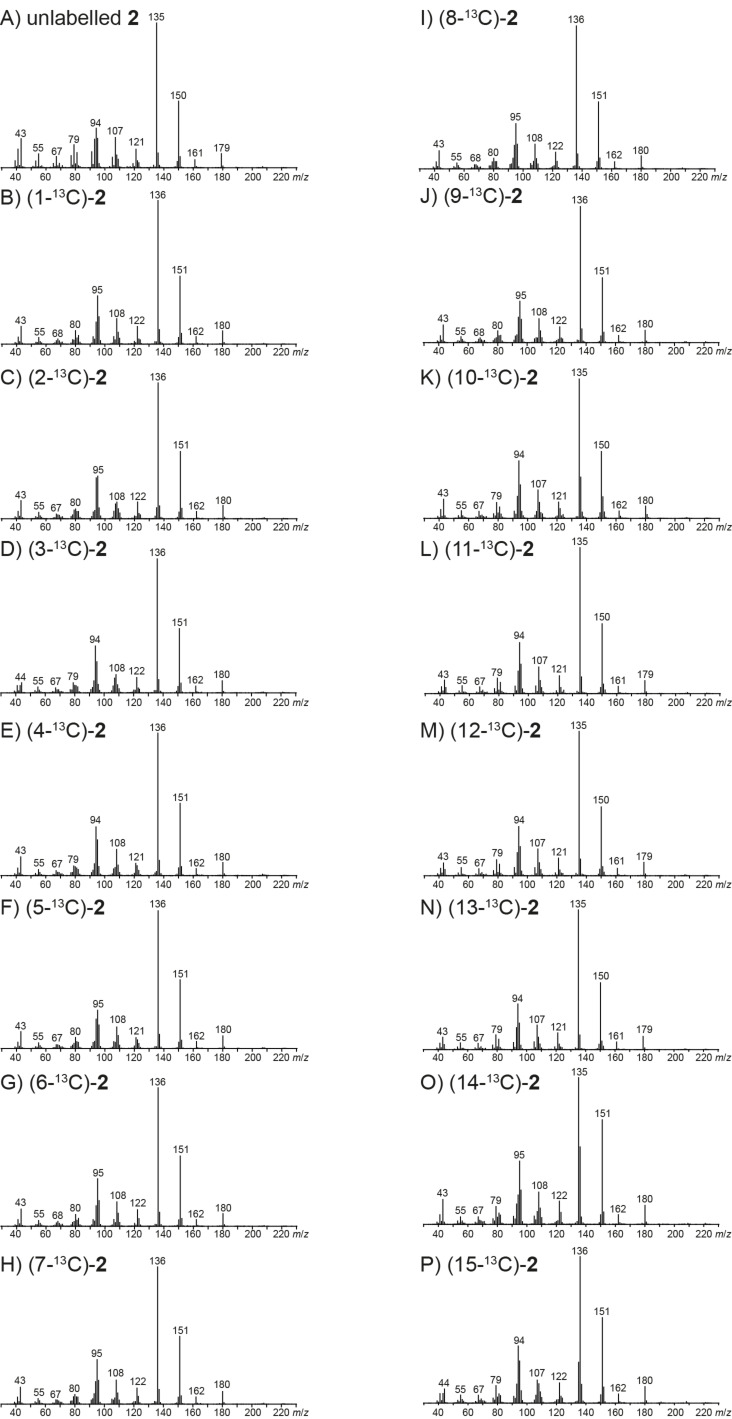
Mass spectra of unlabelled **2** and all fifteen positional isomers of (^13^C_1_)-**2**.

**Scheme 3:**
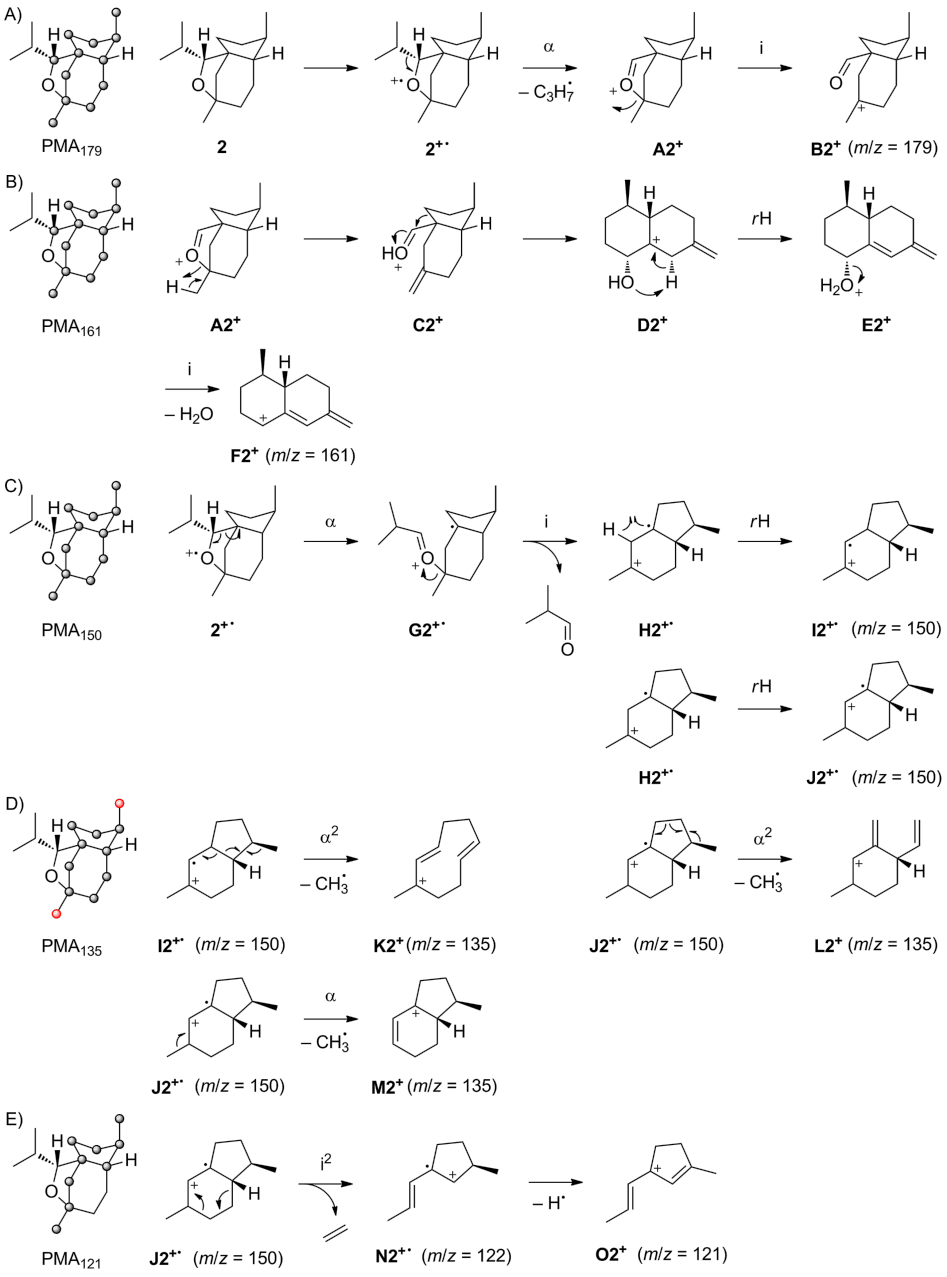
PMAs and EIMS fragmentation mechanisms for the fragment ions A) *m*/*z* = 179, B) *m*/*z* = 161, C) *m*/*z* = 150, D) *m*/*z* = 135 and E) *m*/*z* = 121 of **2**. Black carbons contribute fully and red carbons contribute partially to the formation of a fragment ion. α: α-cleavage, *r*H: hydrogen rearrangement, i: inductive cleavage.

The EI mass spectra of unlabelled **2** and of the fifteen enzymatically obtained isotopomers of (^13^C_1_)-**2** are depicted in Figure 2. As described above for **1**, also for unlabelled **2** the molecular ion [M]^+^ at *m*/*z* = 222 was not detectable. Instead, the highest fragment ion is observed at *m*/*z* = 179, the base peak ion is found at *m*/*z* = 135, and other major fragment ions are at *m*/*z* = 161, 150 and 121. Similar to the situation for **1**, PMA_179_ reveals a formation of *m*/*z* = 179 by loss of the isopropyl group C11–C12–C13 (Scheme 3A). This is explainable by electron impact ionisation of **2** with loss of an electron from an oxygen lone pair to **2^+·^** and subsequent α-cleavage to **A2^+^** that may stabilise by ring opening to **B2^+^**. The PMA_161_ indicates that the fragment ion *m*/*z* = 161 represents the same part of the carbon backbone of **2** as *m*/*z* = 179 (Scheme 3B), while MS^2^ analysis of *m*/*z* = 179 shows that this fragment ion is a precursor of *m*/*z* = 161 (Figure S4, Supporting Information File 1), requiring the neutral loss of water. The relevant fragmentation reaction may proceed by ring opening of **A2^+^** to **C2^+^** followed by a ring expansion to **D2^+^**, a proton transfer to **E2^+^** and loss of water to **F2^+^**. The fragment ion **F2^+^** is structurally the same as **F1^+^** suggested above for *m*/*z* = 161 of **1**. This structural identity of **F1^+^** and **F2^+^** is also reflected by the highly similar MS^2^ spectra of *m*/*z* = 161 for the corvol ethers A and B (Figures S2 and S5, Supporting Information File 1).

The PMA_150_ indicates a clear formation of *m*/*z* = 150 from **2** with loss of the C10–C13 moiety (Scheme 3C). A possible mechanism leading to this fragment ion starts from **2^+·^** that may undergo an α-fragmentation to **G2^+·^**, followed by an inductive cleavage with neutral loss of isobutyraldehyde to **H2^+·^**. This reactive intermediate can stabilise to the conjugated radical cations **I2^+·^** or **J2^+·^** by two alternative hydrogen rearrangements.

PMA_135_ for the base peak ion *m*/*z* = 135 reveals that this fragment ion is produced from C1–C9 plus either C14 or C15 (Scheme 3D). In addition, MS^2^ analysis of *m*/*z* = 150 identifies this fragment ion as a direct precursor for *m*/*z* = 135 by loss of one of the methyl groups C14 or C15 (Figure S6, Supporting Information File 1). Plausible mechanisms include two consecutive or sequential α-fragmentations from **I2^+·^** with loss of C14 to **K2^+^**. Alternatively, two α-fragmentations from **J2^+·^** may yield **L2^+^** that is structurally different from **K2^+^**, but represents the same part of the carbon skeleton of **2** (a similar reaction is also possible from **H2^+^**). The less pronounced loss of C15 is explainable by simple α-fragmentation of **J2^+·^** that results in the allyl cation **M2^+^**.

PMA_121_ (Scheme 3E) points to a formation of the fragment ion at *m*/*z* = 121 by loss of C4–C5 and C10–C13 of **2**, but cleavage of the C4–C5 portion is less clear than that of C10–C13, because the fragment ion *m*/*z* = 122 has a significantly increased intensity in the mass spectra of (4-^13^C)-**2** and (5-^13^C)-**2** in comparison to the mass spectrum of unlabelled **2** (Figure 2). However, MS^2^ analysis of *m*/*z* = 150 shows that this fragment ion is a direct precursor of *m*/*z* = 121. Taken together, these data indicate that loss of C4–C5 from a fragment ion *m*/*z* = 150 is important for the formation of *m*/*z* = 121, but possibly not the only relevant process. A possible mechanism for the fragmentation to *m*/*z* = 121 starts from **J2^+·^** by two inductive or α-cleavages with neutral loss of ethene to **N2^+·^** and subsequent cleavage of a hydrogen, e.g., to **O2^+^** or a similar conjugated cation.

### EIMS fragmentation of *epi*-cubebol

**Figure 3:**
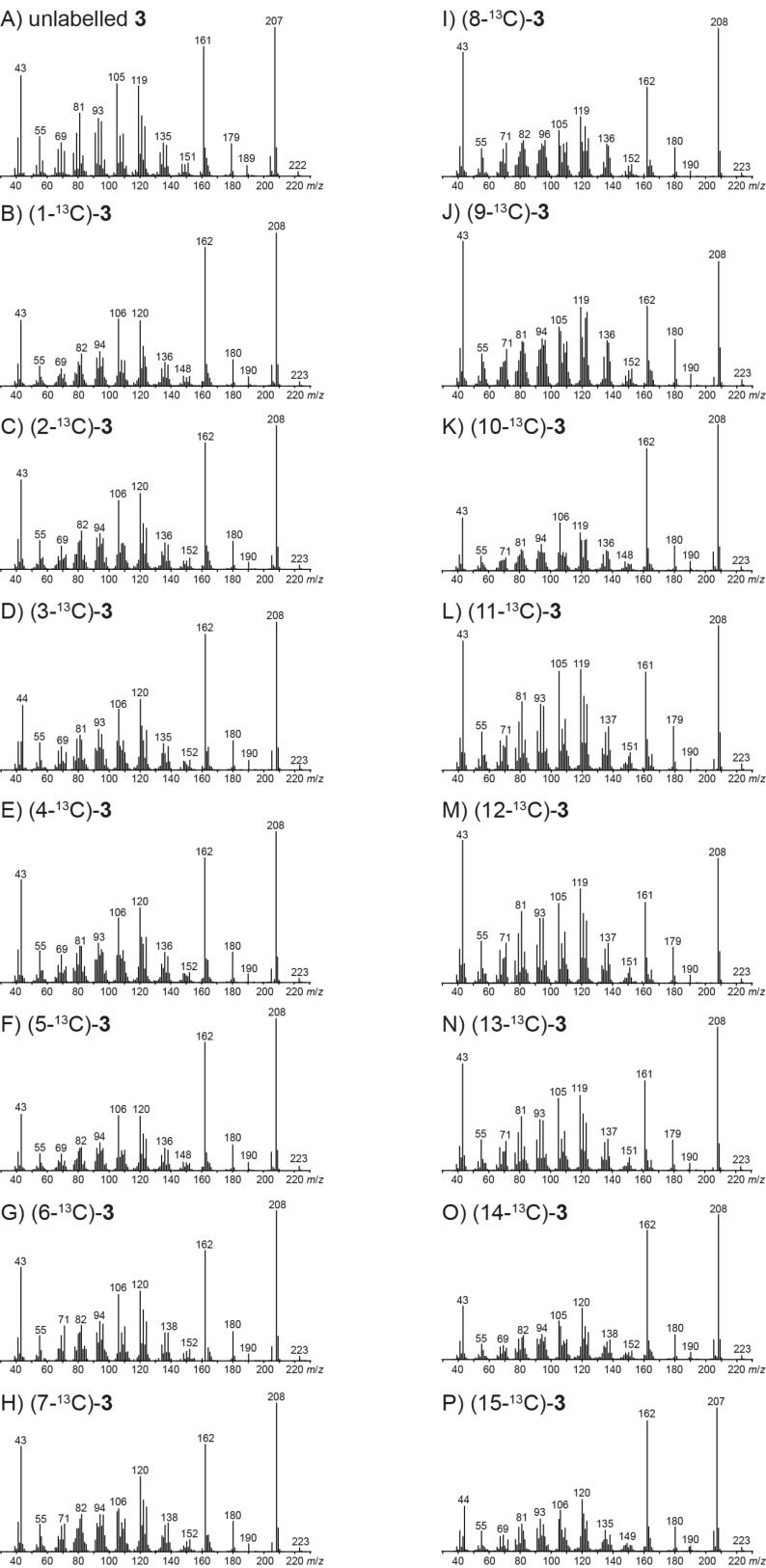
Mass spectra of unlabelled **3** and all fifteen positional isomers of (^13^C_1_)-**3**.

**Scheme 4:**
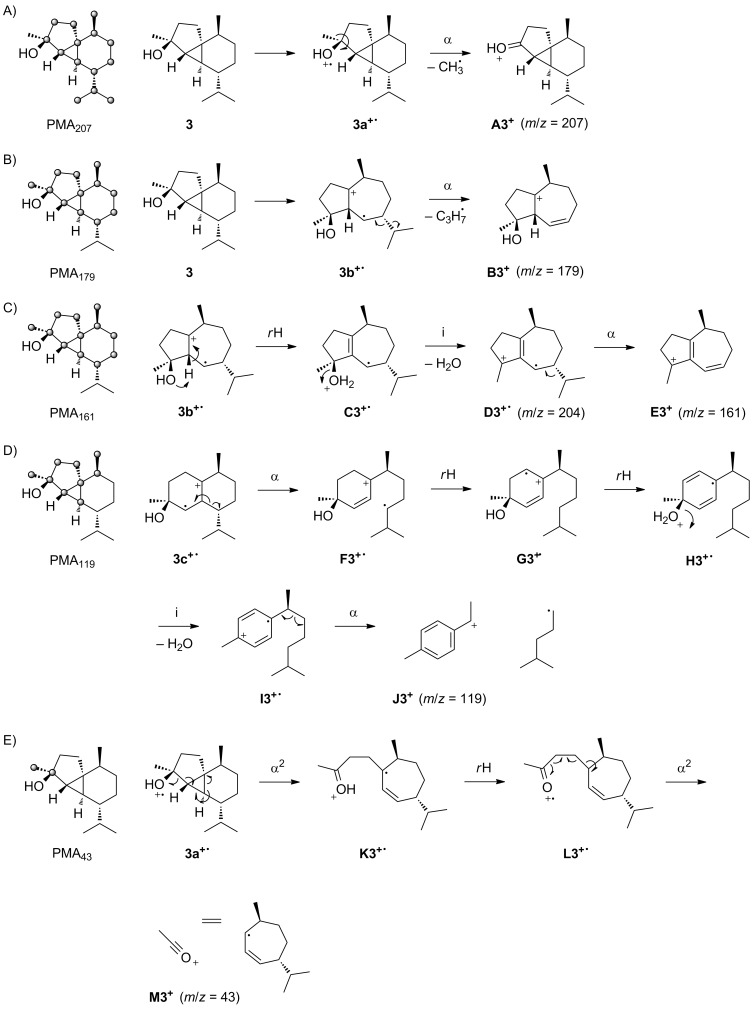
PMAs and EIMS fragmentation mechanisms for the fragment ions A) *m*/*z* = 207, B) *m*/*z* = 179, C) *m*/*z* = 161, D) *m*/*z* = 119 and E) *m*/*z* = 43 of **3**. Black carbons contribute fully and red carbons contribute partially to the formation of a fragment ion. α: α-cleavage, *r*H: hydrogen rearrangement, i: inductive cleavage.

The mass spectra of **3** and the fifteen corresponding isotopomers of (^13^C_1_)-**3** are shown in Figure 3. For the unlabelled compound a small, but clearly visible molecular ion is detected at *m*/*z* = 222. The base peak is recorded at *m*/*z* = 207 and other important fragment ions are observed at *m*/*z* = 179, 161, 119 and 43. As revealed by PMA_207_, the base peak ion in the mass spectrum of **3** is only formed by loss of the methyl group C15, while cleavages of any of the other methyl groups do not contribute to its formation (Scheme 4A). This is easily understood by electron impact ionisation of **3** at the hydroxy function to **3a^+·^**, followed by α-cleavage of C15. PMA_179_ indicates a formation of the fragment ion *m*/*z* = 179 by loss of the isopropyl group C11–C12–C13 of **3** (Scheme 4B). Usually in sesquiterpene alcohols the electron impact ionisation proceeds with loss of an electron from one of the oxygen lone pairs, but in the special case of *epi*-cubebol that contains a 3-membered ring the ionisation step may also proceed with removal of an electron from the energetically high molecular orbitals of the cyclopropane moiety, resulting in **3b^+·^**. Neutral loss of the isopropyl group is then possible by α-cleavage to **B3^+^**. PMA_161_ shows that the fragment ion *m*/*z* = 161 represents the same part of the carbon skeleton of **3** as *m*/*z* = 179 (Scheme 4C), requiring the elimination of water that is plausible starting from **3b^+·^** by a hydrogen rearrangement to **C3^+·^**, neutral loss of water by inductive cleavage to **D3^+·^** and α-cleavage to **E3^+^**. The reverse order of steps for the losses of the isopropyl group and water is also possible, but MS^2^ analyses of *m*/*z* = 204 and *m*/*z* = 179 show that this alternative mechanism is much less pronounced (Figures S7 and S8, Supporting Information File 1).

For PMA_119_ there is not in all fifteen labelling experiments a clear indication, whether or not the respective carbon contributes to the fragment ion *m*/*z* = 119, but the most important mechanism for its formation involves the loss of the C8–C13 fragment of **3** (Scheme 4D). This is explainable by ionisation of **3** to **3c^+·^**, followed by α-cleavage to **F3^+·^**. Two subsequent hydrogen rearrangements result in the conjugated reactive intermediates **G3^+·^** and **H3^+·^**, which may be followed by the elimination of water to **I3^+·^** that upon α-cleavage yields the benzyl cation **J3^+^**. Finally, PMA_43_ reveals a clean formation of the fragment ion *m*/*z* = 43 from C3 and C15 (Scheme 4E). As shown by HRMS analysis, this fragment ion contains oxygen (measured: 43.0183, calculated for [C_2_H_3_O]^+^: 43.0179). The radical cation **3a^+·^** can undergo two sequential or consecutive α-fragmentations to **K3^+·^**. A subsequent hydrogen rearrangement to **L3^+·^** and two more α-cleavages yield the acetylium cation **M3^+^**, ethene and an allyl radical.

### EIMS fragmentation of isodauc-8-en-11-ol

**Figure 4:**
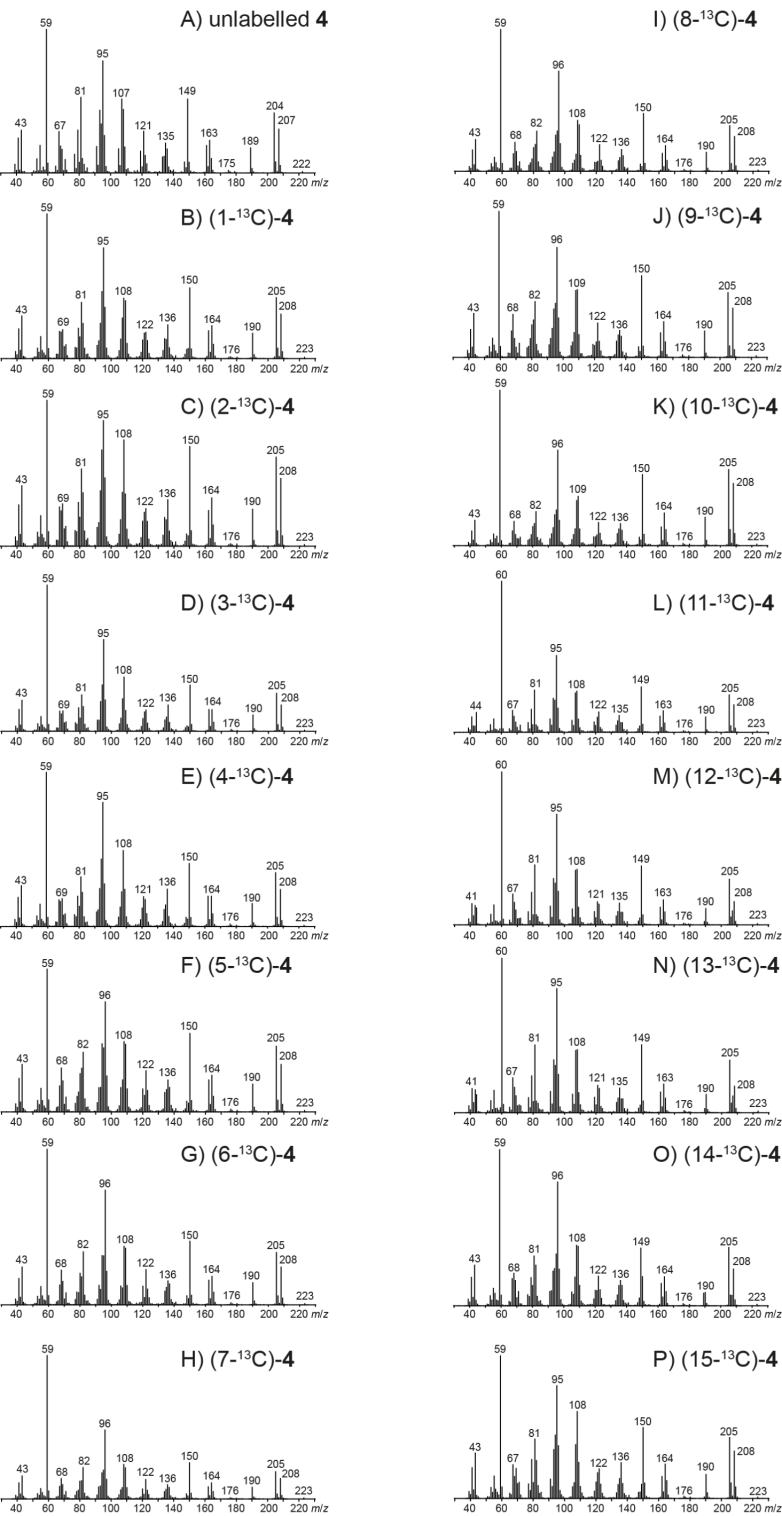
Mass spectra of unlabelled **4** and all fifteen positional isomers of (^13^C_1_)-**4**.

**Scheme 5:**
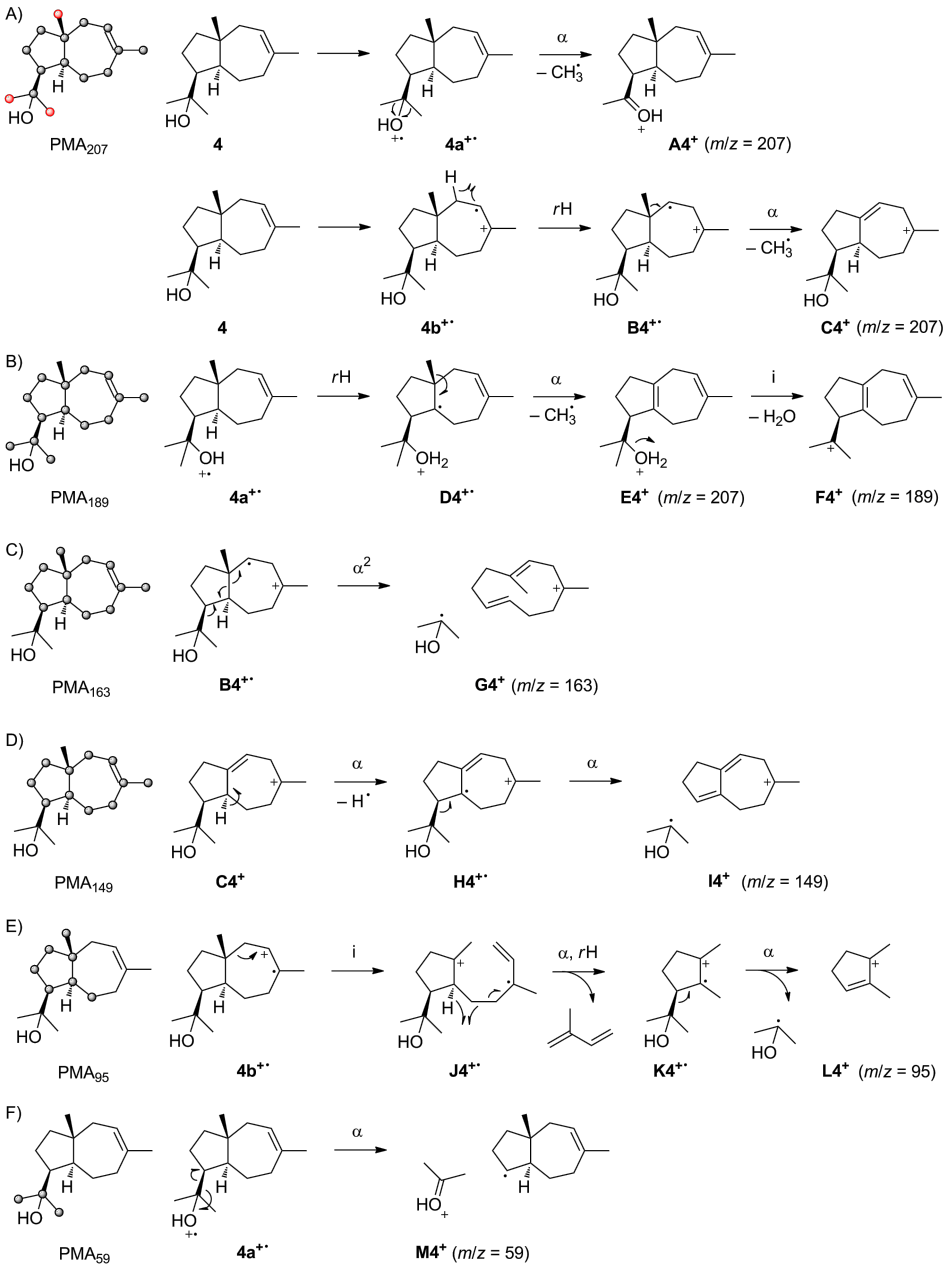
PMAs and EIMS fragmentation mechanisms for the fragment ions A) *m*/*z* = 207, B) *m*/*z* = 189, C) *m*/*z* = 163, D) *m*/*z* = 149, E) *m*/*z* = 95 and F) *m*/*z* = 59 of **4**. Black carbons contribute fully and red carbons contribute partially to the formation of a fragment ion. α: α-cleavage, *r*H: hydrogen rearrangement, i: inductive cleavage.

The mass spectra of unlabelled **4** and its (^13^C_1_)-isotopomers are presented in Figure 4. For the unlabelled compound a small, but visible molecular ion is detected at *m*/*z* = 222. The base peak is observed at *m*/*z* = 59 and other important fragment ions are detected at *m*/*z* = 207, 189, 163, 149 and 95. The PMA_207_ shows that in contrast to the situation for **3** the fragment ion *m*/*z* = 207 arises by cleavage of one of several methyl groups, i.e., either C12, C13 or C14 is lost (Scheme 5A). Electron impact ionisation at the oxygen lone pairs of **4** results in the radical cation **4a^+·^** that can undergo one of two possible α-cleavages with loss of C12 or C13 to yield **A4^+^**. The alternative ionisation of **4** with loss of an electron from the olefinic double bond leads to **4b^+·^** that may react by hydrogen rearrangement to **B4^+·^** and α-cleavage of C14 to **C4^+^**. Interestingly, PMA_189_ demonstrates that the fragment ion *m*/*z* = 189 is formed by loss of water and C14, while cleavage of C12 or C13 is prevented by the elimination of water (Scheme 5B). After ionisation to **4a^+·^**, a hydrogen rearrangement results in **D4^+·^** which enables the loss of C14 by α-cleavage to **E4^+^**. A subsequent inductive cleavage with neutral loss of water yields **F4^+^**. The last two steps of this mechanism may also proceed in reversed order. Both orders of steps are indeed active for this fragmentation mechanism, as indicated by MS^2^ analysis of *m*/*z* = 207 and *m*/*z* = 204 (Figures S9 and S10, Supporting Information File 1).

The PMA_163_ indicates a formation of *m*/*z* = 163 by elimination of the C11–C12–C13 fragment including the alcohol function (Scheme 5C). The best explanation for this finding is a double α-cleavage from **B4^+·^** directly to **G4^+^**. The intensive fragement ion *m*/*z* = 149 requires loss of the hydroxyisopropyl group plus C14, as summarised by PMA_149_ (Scheme 5D). Starting from **C4^+^** in which C14 is already missing, α-cleavage of a hydrogen to the allyl radical **H4^+·^** that induces a second α-cleavage of the hydroxyisopropyl moiety results in **I4^+^**.

Usually, the formation of fragment ions in the low *m*/*z* region is not easily explained, because various multi-step processes lead to these ions. In contrast, PMA_95_ points to a surprisingly clear fragmentation mechanism, indicating that the fragment ion *m*/*z* = 95 is made up from C5–C10 + C14 of **4** (Scheme 5E). A plausible mechanism starts from **4b^+·^** that results in **J4^+·^** by inductive cleavage, followed by a neutral loss of isoprene to **K4^+·^**. The α-cleavage of **J4^+·^** would yield a primary radical, but this can immediately be stabilised if the α-cleavage is coupled with a hydrogen rearrangement to generate the conjugated radical cation **K4^+·^**. Another α-cleavage of the hydroxyisopropyl group results in **L4^+^**. Finally, the PMA_59_ demonstrates formation of this fragment ion from the hydroxyisopropyl group which is possible by a single α-cleavage from **4a^+·^** to **M4^+^** (Scheme 5F).

## Conclusion

Isotopic labelling experiments continue to be a valuable source of information for many questions in natural product chemistry [23]. Many recent examples have shown how isotopic labelling experiments can unravel the complex and sometimes surprising cyclisation cascades that are catalysed by terpene cyclases in the conversion of simple linear substrates to polycyclic terpenes with usually several stereocentres [24–28]. We have demonstrated here, how isotopic labelling experiments can be used to investigate the EIMS fragmentation mechanisms of structurally complex sesquiterpenes. This field was initiated in the 1960s with main contributions by Djerassi, but since that time, not much knowledge has been added. One of the main problems is certainly the accessibility of isotopically labelled terpenes. We have solved this problem by our recent synthesis of all fifteen isotopomers of (^13^C_1_)FPP [16] that can now be used for enzymatic conversions into sesquiterpenes, which allows for various enzyme mechanistic experiments and, as we have shown here, for investigations on the EIMS fragmentation mechanisms. We will continue to report on other mechanistic problems of terpene chemistry by use of the (^13^C_1_)FPPs in due course.

## Experimental

### GC–MS and GC–QTOF MS analysis

GC–MS analyses were performed using a 7890B gas chromatograph connected to a 5977A inert mass detector (Agilent). The GC was equipped with a HP5-MS fused silica capillary column (30 m, 0.25 mm i. d., 0.50 μm film). The instrumental parameters of the GC were (1) inlet pressure: 77.1 kPa, He 23.3 mL min^−1^, (2) injection volume: 2 μL, (3) split mode (10:1 to 50:1, 60 s valve time), (4) carrier gas: He 1 mL min^−1^, (5) transfer line: 250 °C, and (6) electron energy: 70 eV. The temperature program of GC was set to: 5 min at 50 °C, then increasing by 10 °C min^−1^ to 320 °C, followed by 5 min at 320 °C.

GC–HRMS analyses were carried out with a 7890B gas chromatograph connected to a 7200 accurate mass QTOF mass detector (Agilent) equipped with a HP5-MS fused silica capillary column (30 m, 0.25 mm i. d., 0.50 μm film). The instrumental parameters were (1) inlet pressure: 83.2 kPa, He 24.6 mL min^−1^, (2) injection volume: 2 μL, (3) split mode (10:1 to 50:1, 60 s valve time), (4) carrier gas: He 1 mL min^−1^, (5) transfer line: 250 °C, (6) electron energy 70 eV, (7) collision cell gas flow: 1 mL min^−1^ N_2_, (8) collision energy: 15 V, and (9) MS 1 scan resolution mode: narrow (*m*/*z* +/− 0.5). The temperature program of GC was set to: 5 min at 50 °C, then increasing by 10 °C min^−1^ to 320 °C, followed by 5 min at 320 °C.

### Incubation experiments with labelled (^13^C_1_)FPPs

*E. coli* BL 21 was transformed with the appropriate expression plasmid for corvol ether synthase, *epi*-cubebol synthase or isodauc-8-en-11-ol synthase [18–19,21]. The transformants were used to inoculate a 20 mL 2YT liquid preculture (tryptone 16 g, yeast extract 10 g, NaCl 5 g, water 1 L) containing kanamycin (50 mg/L) that was grown overnight. The next morning the preculture was used to inoculate an expression culture (2YT, 1 L) containing kanamycin (50 mg/L). The cells were grown to an OD_600_ = 0.4 at 37 °C and 160 rpm. After cooling of the culture to 18 °C for 45 minutes, IPTG (0.4 mM) was added. The culture was incubated at 18 °C and 160 rpm overnight for protein expression. Harvesting of *E. coli* cells by centrifugation at 4 °C and 3600 rpm for 45 minutes, resuspension in 20 mL binding buffer (20 mM Na_2_HPO_4_, 0.5 M NaCl, 20 mM imidazole, 1 mM MgCl_2_, pH 7.0) and cell disruption by ultra-sonication on ice for 4 × 60 s followed by centrifugation at 4 °C and 11000 rpm, yielded in the soluble enzyme fraction. Protein purification was performed by Ni^2+^-NTA affinity chromatography with Ni^2+^-NTA superflow (Novagen) using binding buffer and elution buffer (20 mM Na_2_HPO_4_, 0.5 M NaCl, 0.5 M imidazole, 1 mM MgCl_2_, pH 7.0). Incubation experiments were performed with the pure protein fractions (checked by SDS-PAGE) and all fifteen isotopomers of farnesyl diphosphate. Incubation experiments were carried out using 1 mL of the enzyme fraction and 1 mL of a solution of the isotopomer of (^13^C_1_)FPP (0.2 mg/mL in H_2_O) for 3 h at 28 °C. The reaction mixtures were extracted with *n*-hexane (300 μL) and analyzed by GC–MS and GC–QTOF MS.

## Supporting Information

File 1 HRMS spectra.
