# Supplementary material for: The EIMS fragmentation mechanisms of the sesquiterpenes corvol ethers A and B, epi-cubebol and isodauc-8-en-11-ol
Source: Beilstein J Org Chem. 2016 Jul 5;12:1380–94. doi: 10.3762/bjoc.12.132 (PMC4979868; doi:10.3762/bjoc.12.132)
Supplement: File 1 — HRMS spectra. [file Beilstein_J_Org_Chem-12-1380-s001.pdf]

Supporting Information

for

The EIMS fragmentation mechanisms of the sesquiterpenes corvol ethers A and B, *epi*-cubebol and isodauc-8-en-11-ol

Patrick Rabe and Jeroen S. Dickschat*

Address: Kekulé-Institute of Organic Chemistry and Biochemistry, University of Bonn, Gerhard-Domagk-Straße 1, 53121 Bonn, Germany

Email: Jeroen S. Dickschat - dickschat@uni-bonn.de

* Corresponding author

HRMS spectra

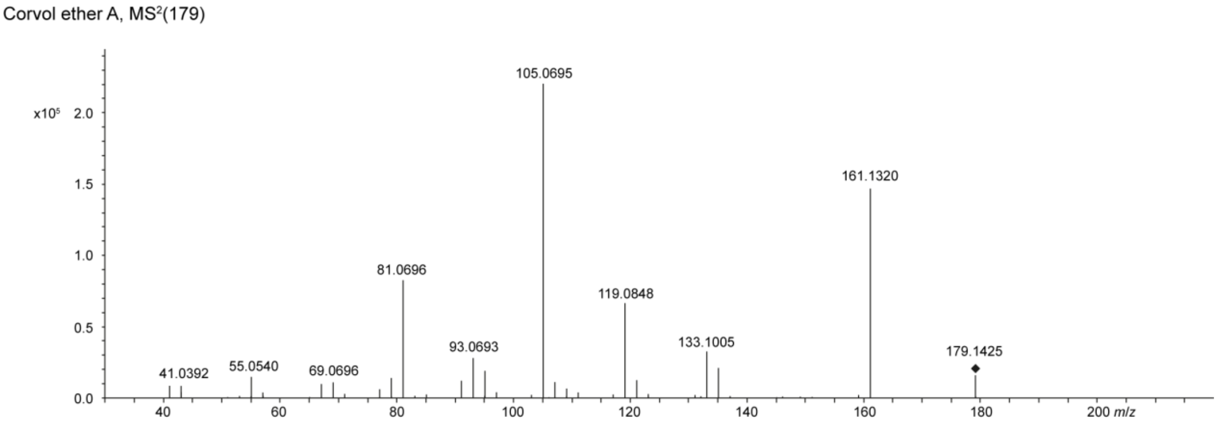

Figure S1: MS^2 for *m*/*z* = 179 of **1**.

Table S1: Elemental compositions of fragment ions in Figure S1.

molecular formula	*m*/*z* found	*m*/*z* calculated	Δ (ppm)
$[C_{12}H_{19}O]^+$	179.1425	179.1430	–2.8
$[C_{12}H_{17}]^+$	161.1320	161.1325	–3.1
$[C_{10}H_{13}]^+$	133.1005	133.1012	+5.3
$[C_9H_{11}]^+$	119.0848	119.0855	+5.9
$[C_8H_9]^+$	105.0695	105.0699	+3.8
$[C_7H_9]^+$	93.0693	93.0699	+3.2
$[C_6H_9]^+$	81.0696	81.0699	+3.7
$[C_5H_9]^+$	69.0696	69.0699	+4.3
$[C_4H_7]^+$	55.0540	55.0542	+3.6
$[C_3H_5]^+$	41.0392	41.0386	+14.6

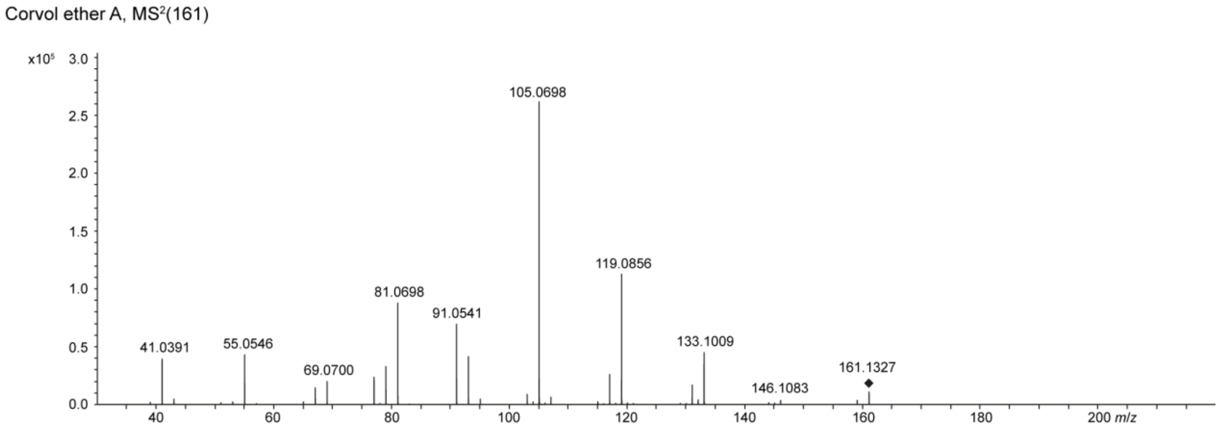

Figure S2: MS^2 for *m*/*z* = 161 of **1**.

Table S2: Elemental compositions of fragment ions in Figure S2.

**molecular formula**	***m*/*z* found**	***m*/*z* calculated**	**Δ (ppm)**
$[C_{12}H_{17}]^+$	161.1327	161.1325	+1.2
$[C_{11}H_{14}]^+$	146.1083	146.1090	–4.8
$[C_{10}H_{13}]^+$	133.1009	133.1012	–2.3
$[C_9H_{11}]^+$	119.0856	119.0855	+0.8
$[C_8H_9]^+$	105.0698	105.0699	–1.0
$[C_7H_7]^+$	91.0541	91.0542	–1.1
$[C_6H_9]^+$	81.0698	81.0699	–1.2
$[C_5H_9]^+$	69.0700	69.0699	+1.4
$[C_4H_7]^+$	55.0546	55.0542	+7.3
$[C_3H_5]^+$	41.0391	41.0386	+12.2

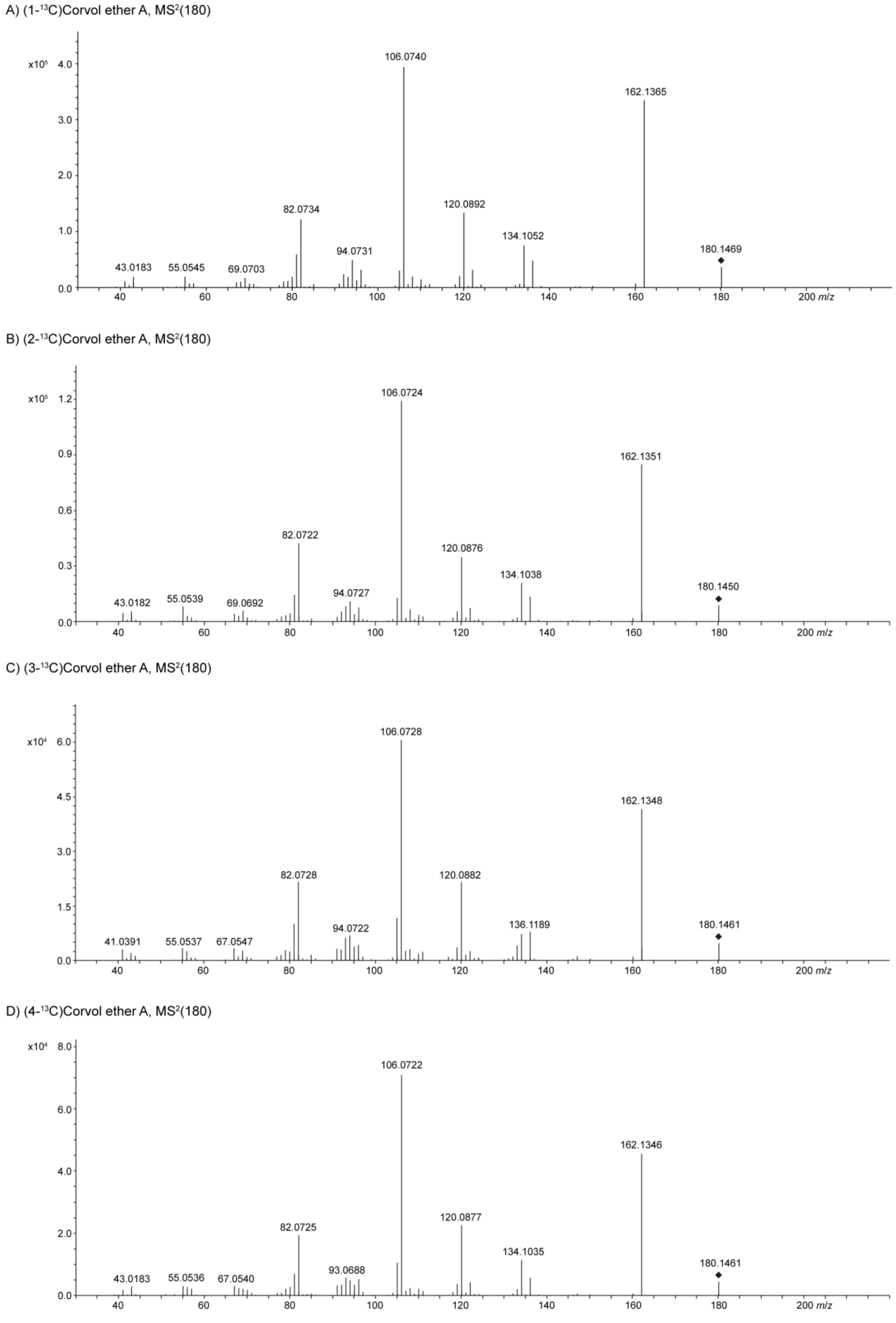

Figure S3: MS^2 for *m*/*z* = 180 of A) (1-^{13}C)-**1**, B) (2-^{13}C)-**1**, C) (3-^{13}C)-**1**, D) (4-^{13}C)-**1**.

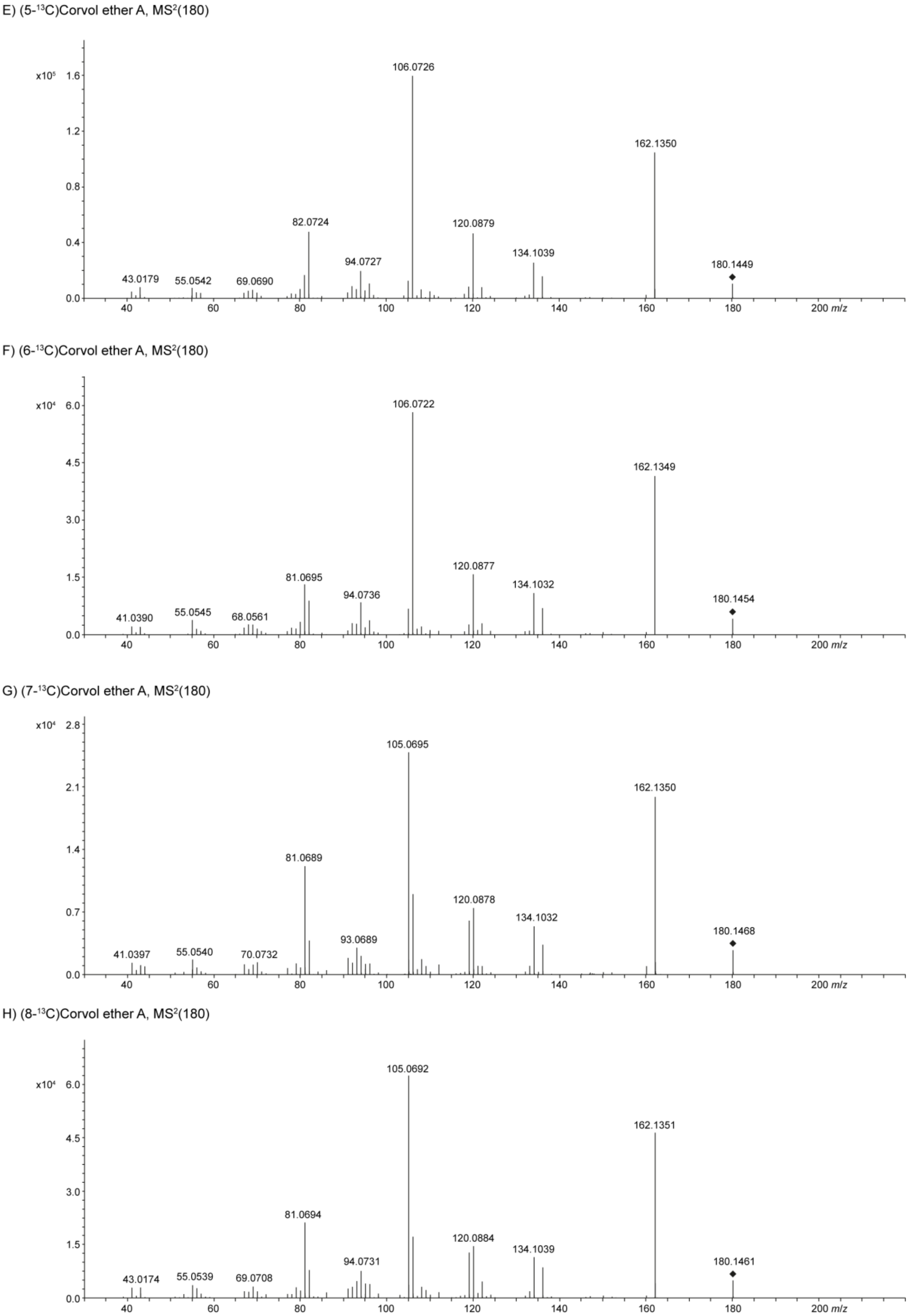

Figure S3: MS^2 for *m*/*z* = 180 of E) (5-^{13}C)-**1**, F) (6-^{13}C)-**1**, G) (7-^{13}C)-**1**, H) (8-^{13}C)-**1**.

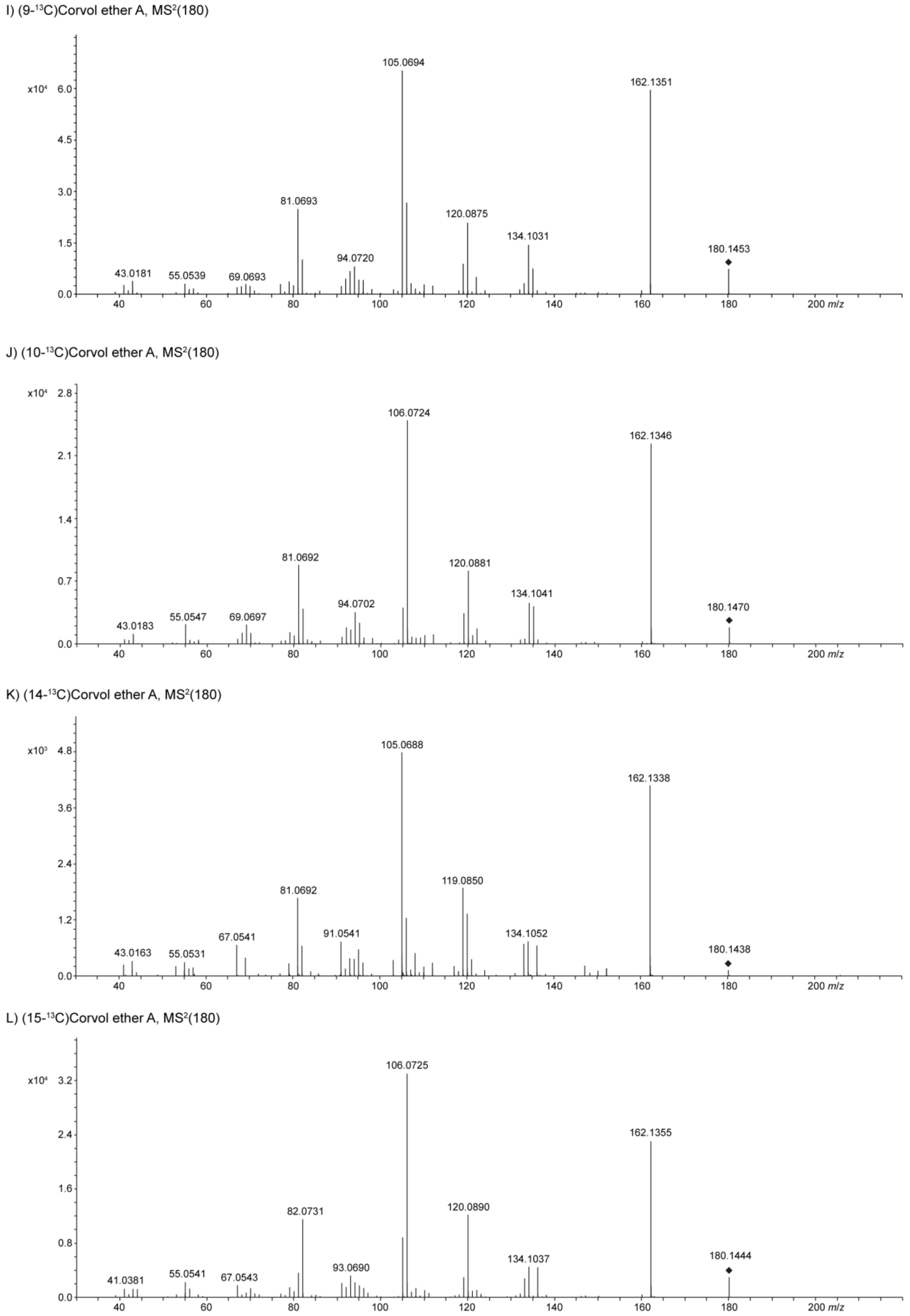

Figure S3: MS^2 for *m/z* = 180 of I) ($9\text{-}^{13}C$)-**1**, J) ($10\text{-}^{13}C$)-**1**, K) ($14\text{-}^{13}C$)-**1**, L) ($15\text{-}^{13}C$)-**1**.

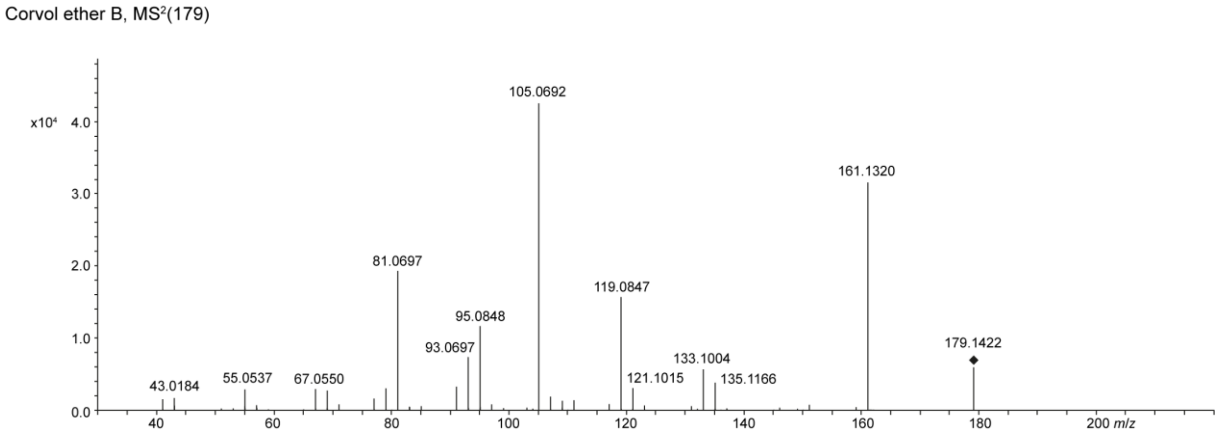

Figure S4: MS^2 for *m*/*z* = 179 of **2**.

Table S3: Elemental compositions of fragment ions in Figure S4.

**molecular formula**	***m*/*z* found**	***m*/*z* calculated**	**Δ (ppm)**
$[C_{12}H_{19}O]^+$	179.1422	179.1430	–4.5
$[C_{12}H_{17}]^+$	161.1320	161.1325	–3.1
$[C_{10}H_{13}]^+$	133.1004	133.1012	–6.0
$[C_9H_{11}]^+$	119.0847	119.0855	–6.7
$[C_8H_9]^+$	105.0692	105.0699	–6.7
$[C_7H_{11}]^+$	95.0848	95.0855	–7.4
$[C_6H_9]^+$	81.0697	81.0699	–2.5
$[C_5H_7]^+$	67.0550	67.0542	+11.9
$[C_4H_7]^+$	55.0540	55.0542	–3.6
$[C_2H_3O]^+$	43.0184	43.0179	+11.6

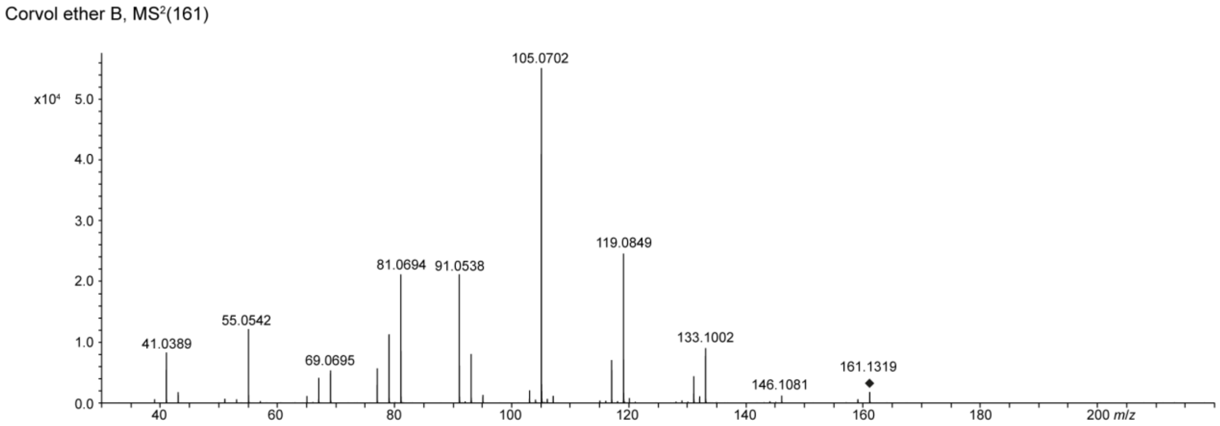

Figure S5: MS^2 for *m*/*z* = 161 of **2**.

Table S4: Elemental compositions of fragment ions in Figure S5.

molecular formula	*m*/*z* found	*m*/*z* calculated	Δ (ppm)
$[C_{12}H_{17}]^+$	161.1319	161.1325	–3.7
$[C_{10}H_{13}]^+$	133.1002	133.1012	–7.5
$[C_9H_{11}]^+$	119.0849	119.0855	–5.0
$[C_8H_9]^+$	105.0702	105.0699	+2.9
$[C_7H_7]^+$	91.0538	91.0542	–4.4
$[C_6H_9]^+$	81.0694	81.0699	–6.2
$[C_5H_9]^+$	69.0695	69.0699	–5.8
$[C_4H_7]^+$	55.0542	55.0542	0.0
$[C_3H_5]^+$	41.0389	41.0386	+7.3

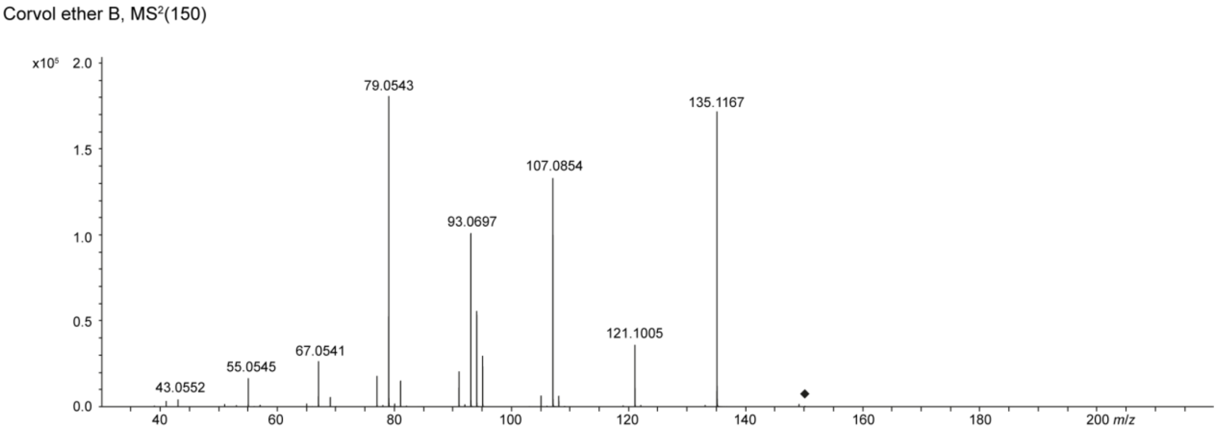

Figure S6: MS^2 for *m*/*z* = 150 of **2**.

Table S5: Elemental compositions of fragment ions in Figure S6.

**molecular formula**	***m*/*z* found**	***m*/*z* calculated**	**Δ (ppm)**
$[C_{10}H_{15}]^+$	135.1167	135.1168	–0.7
$[C_9H_{13}]^+$	121.1005	121.1012	–5.8
$[C_8H_{11}]^+$	107.0854	107.0855	–0.9
$[C_7H_9]^+$	93.0697	93.0699	–2.1
$[C_6H_7]^+$	79.0543	79.0542	+1.2
$[C_5H_7]^+$	67.0541	67.0542	–1.5
$[C_4H_7]^+$	55.0545	55.0542	+5.4
$[C_3H_7]^+$	43.0552	43.0542	+23.2

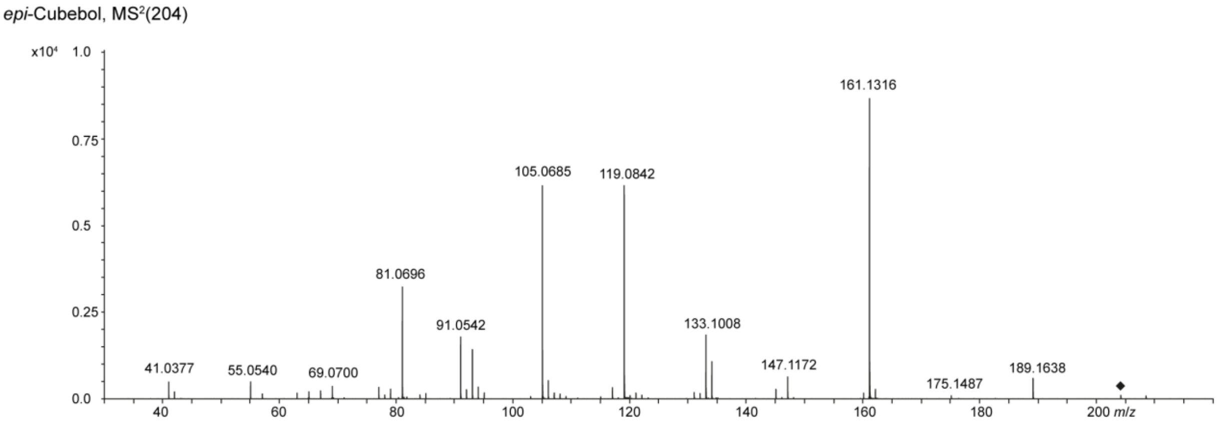

Figure S7: MS^2 for *m*/*z* = 204 of **3**.

Table S6: Elemental compositions of fragment ions in Figure S7.

molecular formula	*m*/*z* found	*m*/*z* calculated	Δ (ppm)
$[C_{14}H_{21}]^+$	189.1638	189.1638	0.0
$[C_{13}H_{19}]^+$	175.1487	175.1482	+2.9
$[C_{12}H_{17}]^+$	161.1316	161.1325	–5.6
$[C_{11}H_{15}]^+$	147.1172	147.1168	+2.7
$[C_{10}H_{13}]^+$	133.1008	133.1012	–3.0
$[C_9H_{11}]^+$	119.0842	119.0855	–10.9
$[C_8H_9]^+$	105.0685	105.0699	–13.3
$[C_7H_7]^+$	91.0542	91.0542	0.0
$[C_6H_9]^+$	81.0696	81.0699	–3.7
$[C_5H_9]^+$	69.0700	69.0699	+1.4
$[C_4H_7]^+$	55.0540	55.0542	–3.6
$[C_3H_5]^+$	41.0377	41.0386	–21.9

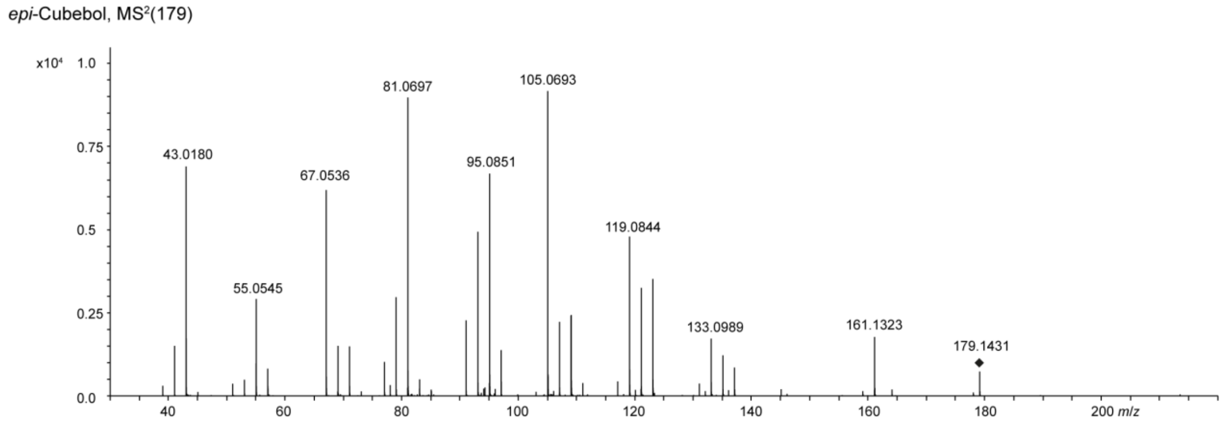

Figure S8: MS^2 for *m*/*z* = 179 of **3**.

Table S7: Elemental compositions of fragment ions in Figure S8.

molecular formula	***m*/*z* found**	***m*/*z* calculated**	**Δ (ppm)**
$[C_{12}H_{19}O]^+$	179.1431	179.1430	+0.6
$[C_{12}H_{17}]^+$	161.1323	161.1325	–1.2
$[C_{10}H_{13}]^+$	133.1008	133.1012	–3.0
$[C_9H_{11}]^+$	119.0844	119.0855	–9.2
$[C_8H_9]^+$	105.0693	105.0699	–5.7
$[C_7H_{11}]^+$	95.0851	95.0855	–4.2
$[C_6H_9]^+$	81.0697	81.0699	–2.5
$[C_5H_7]^+$	67.0536	67.0542	–8.9
$[C_4H_7]^+$	55.0545	55.0542	+5.4
$[C_2H_3O]^+$	43.0180	43.0179	+2.3

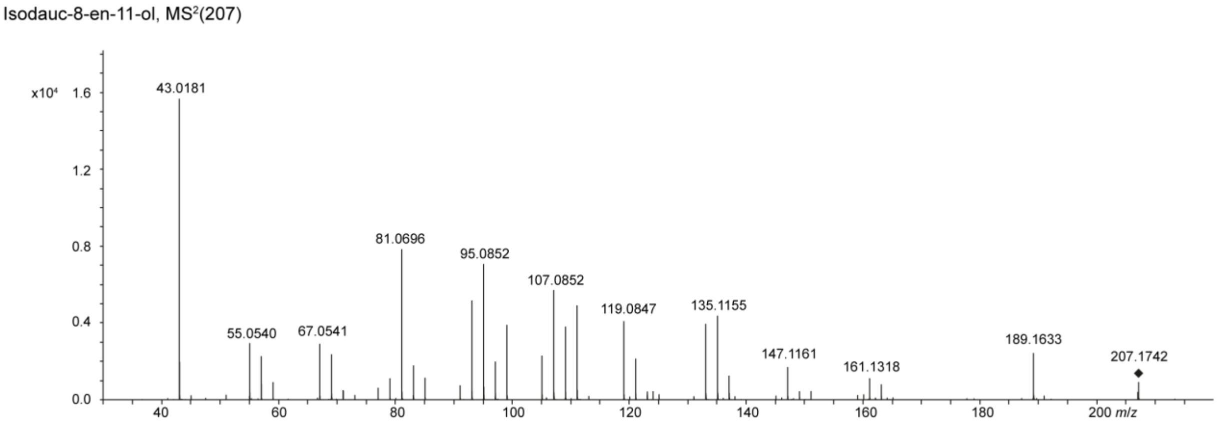

Figure S9: MS^2 for *m*/*z* = 207 of **4**.

Table S8: Elemental compositions of fragment ions in Figure S9.

molecular formula	*m*/*z* found	*m*/*z* calculated	Δ (ppm)
$[C_{14}H_{23}O]^+$	207.1742	207.1743	–0.5
$[C_{14}H_{21}]^+$	189.1633	189.1638	–2.6
$[C_{12}H_{17}]^+$	161.1318	161.1325	–4.3
$[C_{11}H_{15}]^+$	147.1161	147.1168	–4.8
$[C_{10}H_{15}]^+$	135.1155	135.1168	–9.6
$[C_9H_{11}]^+$	119.0847	119.0855	–6.7
$[C_8H_{11}]^+$	107.0852	107.0855	–2.8
$[C_7H_{11}]^+$	95.0852	95.0855	–3.2
$[C_6H_9]^+$	81.0696	81.0699	–3.7
$[C_5H_7]^+$	67.0541	67.0542	–1.5
$[C_4H_7]^+$	55.0540	55.0542	–3.6
$[C_2H_3O]^+$	43.0181	43.0178	+7.0

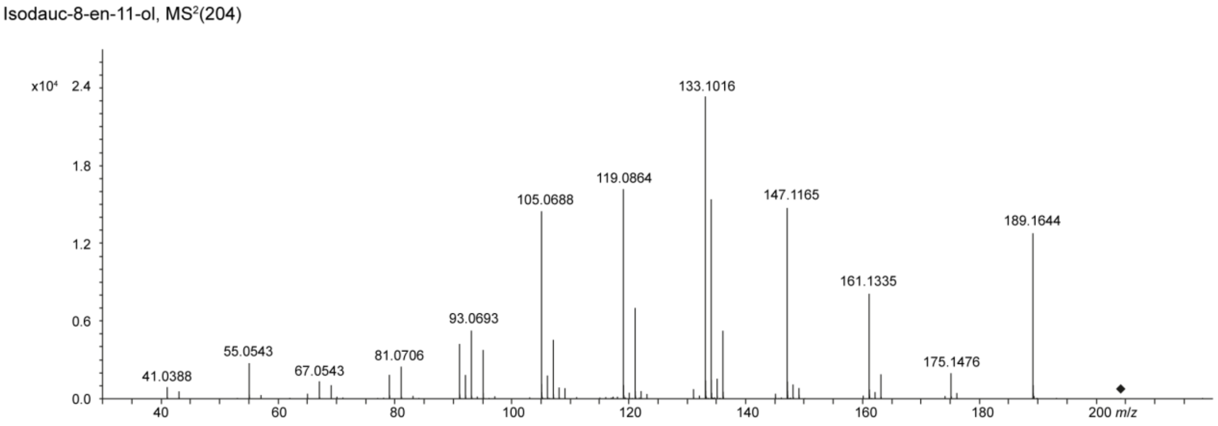

Figure S10: MS^2 for m/z = 204 of **4**.

Table S9: Elemental compositions of fragment ions in Figure S10.

molecular formula	*m/z* found	*m/z* calculated	Δ (ppm)
$[C_{14}H_{21}]^+$	189.1644	189.1638	+3.2
$[C_{13}H_{19}]^+$	175.1476	175.1482	–3.4
$[C_{12}H_{17}]^+$	161.1335	161.1325	+6.2
$[C_{11}H_{15}]^+$	147.1165	147.1168	–2.4
$[C_{10}H_{13}]^+$	133.1016	133.1012	+3.0
$[C_9H_{11}]^+$	119.0864	119.0855	+7.6
$[C_8H_9]^+$	105.0688	105.0699	–10.5
$[C_7H_9]^+$	93.0693	93.0699	–6.4
$[C_6H_9]^+$	81.0706	81.0699	+8.6
$[C_5H_7]^+$	67.0543	67.0542	+1.5
$[C_4H_7]^+$	55.0543	55.0542	+1.8
$[C_3H_5]^+$	41.0388	41.0386	+4.9
